# B4GALT1 promotes immune escape by regulating the expression of PD-L1 at multiple levels in lung adenocarcinoma

**DOI:** 10.1186/s13046-023-02711-3

**Published:** 2023-06-12

**Authors:** Yanan Cui, Jun Li, Pengpeng Zhang, Dandan Yin, Ziyu Wang, Jiali Dai, Wei Wang, Erbao Zhang, Renhua Guo

**Affiliations:** 1 Department of Oncology, First Affiliated Hospital of Nanjing Medical University, Nanjing, Jiangsu P. R. China; 2 Department of Thoracic Surgery, First Affiliated Hospital of Nanjing Medical University, Nanjing, Jiangsu P. R. China; 3 Clinical Research Center, The Second Hospital of Nanjing, Nanjing University of Chinese Medicine, Zhong Fu Road, Gulou District, Nanjing, Jiangsu 210003 P. R. China; 4 Department of Pathology, Nanjing Drum Tower Hospital, The Affiliated Hospital of Nanjing University Medical School, Nanjing, China; 5 Department of Epidemiology, Center for Global Health, School of Public Health, Nanjing Medical University, Nanjing, 211166 China; 6 Jiangsu Key Lab of Cancer Biomarkers, Prevention and Treatment, Collaborative Innovation Center for Cancer Medicine, Nanjing Medical University, Nanjing, 211166 China

**Keywords:** Early-stage lung adenocarcinoma, N-linked glycosylation, PD-L1, Tumor microenvironment

## Abstract

**Background:**

Invasive adenocarcinoma (IAC), which is typically preceded by minimally invasive adenocarcinoma (MIA), is the dominant pathological subtype of early-stage lung adenocarcinoma (LUAD). Identifying the molecular events underlying the progression from MIA to IAC may provide a crucial perspective and boost the exploration of novel strategies for early-stage LUAD diagnosis and treatment.

**Methods:**

Transcriptome sequencing of four pairs of MIA and IAC tumours obtained from four multiple primary lung cancer patients was performed to screen out beta-1,4-galactosyltransferase1 (*B4GALT1*). Function and mechanism experiments in *vitro* and in *vivo* were performed to explore the regulatory mechanism of *B4GALT1*-mediated immune evasion by regulating programmed cell death ligand 1 (PD-L1).

**Results:**

*B4GALT1*, a key gene involved in N-glycan biosynthesis, was highly expressed in IAC samples. Further experiments revealed that *B4GALT1* regulated LUAD cell proliferation and invasion both in vitro and in vivo and was related to the impaired antitumour capacity of CD8 + T cells. Mechanistically, *B4GALT1* directly mediates the N-linked glycosylation of PD-L1 protein, thus preventing PD-L1 degradation at the posttranscriptional level. In addition, *B4GALT1* stabilized the TAZ protein via glycosylation, which activated *CD274* at the transcriptional level. These factors lead to lung cancer immune escape. Importantly, inhibition of *B4GALT1* increased CD8 + T-cell abundance and activity and enhanced the antitumour immunity of anti-PD-1 therapy in vivo.

**Conclusion:**

*B4GALT1* is a critical molecule in the development of early-stage LUAD and may be a novel target for LUAD intervention and immunotherapy.

**Supplementary Information:**

The online version contains supplementary material available at 10.1186/s13046-023-02711-3.

## Background

As lung cancer screening has become widespread and computed tomography (CT) has been performed more often in clinical practice, the number of lung nodules detected has risen considerably, many of which are adenocarcinoma in situ (AIS), minimally invasive adenocarcinoma (MIA), and invasive adenocarcinoma (IAC) [1, 2]. After surgical resection, the 5-year survival rates for AIS and MIA are nearly 100%, and the recurrence rates are 0%, whereas IAC does not have such a good survival benefit [3]. Several professional societies have revised the classification of lung adenocarcinoma (LUAD) and its precursors, including a stepwise evolutionary spectrum from atypical adenomatous hyperplasia (AAH) to AIS, MIA, and eventually IAC [4–6]. Previous studies have described the genomic, immune, and metabolic landscapes of AIS, MIA, and IAC, which may assist in early cancer detection and prevention [7–11]. However, there remains a lack of deep insight into the precise molecular events underlying the progression trajectory of early-stage LUAD from MIA to IAC.

Multiple primary lung cancer (MPLC) refers to the presence of several tumours of independent origin in the same patient, often in the form of multiple pulmonary nodules, mostly MIA or IAC. The comparison of different lesions in the same tumour niche can exclude the bias caused by the differences in patients’ genetic backgrounds, allowing a deeper understanding of the internal molecular alterations of tumours in the progression from MIA to AIS. Therefore, in this study, we specifically collected MPLC patients with tumours of different invasion states for pairwise comparison. Based on this unique MPLC model, we determined that beta-1,4-galactosyltransferase 1 (*B4GALT1*), an N-glycan synthesis-related gene, is essential for the progression of early-stage LUAD.

Aberrant N-linked glycosylation is common in tumorigenesis, including lung cancer [12, 13]. N-glycosylation patterns contribute to the stability and activity of numerous oncogenic proteins, which could promote cancer progression [14–16]. *B4GALT1* participates in the formation of N-glycosylation by transferring β-1,4-chain galactose to acceptor sugars, and accumulating evidence has implicated this protein in tumour biology and progression [17]. For instance, *B4GALT1* expression has been shown to be associated with a worse prognosis in bladder cancer, clear cell renal cell carcinomas, and pancreatic ductal adenocarcinomas [18–20]. Nevertheless, the role of *B4GALT1* in lung cancer and its underlying molecular mechanism are far from clear. In this work, we aimed to uncover the biological function of *B4GALT1* in the tumorigenesis of early-stage LUAD and investigated its role in immune evasion.

## Methods

### Clinical sample collection and RNA-sequencing

The four MPLC patients and their clinical data were collected from Jiangsu Provincial People’s Hospital. Tumour tissues were obtained for research purposes from the surgical specimens on the day of surgery, and the tumour tissues were identified by the pathologist as MIA or IAC. These tumour samples were sent to Oncocares Inc. (Suzhou, China) for RNA sequencing. We applied the R “umap” package to perform the uniform manifold approximation and projection (UMAP) analysis. Kyoto Encyclopedia of Genes and Genomes (KEGG) and Gene Ontology (GO) analyses were conducted with the “enrichR” (R package). The abundance levels of 22 immune cells were determined using CIBERSORT (https://cibersort.stanford.edu).

### Bioinformatic analysis of public datasets

The Cancer Genome Atlas (TCGA) RAN-sequencing data and patient information were downloaded from the UCSC Xena dataset (http://xena.ucsc.edu/welcome-to-ucsc-xena/), and TCGA protein expression data were obtained from The Cancer Proteome Atlas dataset (http://tcpaportal.org/tcpa/index.html). The GSE166720 and GSE31210 datasets were obtained from the Gene Expression Omnibus (GEO) database (http://www.ncbi.nlm.nih.gov/geo/). The Cancer Cell Line Encyclopedia (CCLE) mRNA data were obtained from http://www.broadinstitute.org/ccle. Survival analysis was performed using the “survival” and “survminer” packages in R. The cytotoxic T-lymphocyte (CTL) level was calculated based on the average expression levels of *CD8A*, granzyme A (*GZMA*), *CD8B*, granzyme B (*GZMB*), and perforin 1. The Tumour Immune Dysfunction and Exclusion (TIDE) score was calculated using online software (http://tide.dfci.harvard.edu). The detailed method is described in the TIDE algorithm [21].

### Cell culture

We purchased two LUAD cell lines (A549 and PC9) from the Institute of Biochemistry and Cell Biology of the Chinese Academy of Sciences (Shanghai, China). The medium consisted of DMEM or RPMI 1640 containing 10% foetal bovine serum (FBS) and 1% antibiotics (100 U/ml penicillin and 100 mg/ml streptomycin) and was used to maintain the PC9 or A549 cells, respectively. Lewis cells (LLCs) were purchased from Zhong Qiao Xin Zhou Biotechnology Co., Ltd (Shanghai, China) and maintained in DMEM.

### RNA extraction and qRT‒PCR analyses

Total RNA was extracted using TRIzol reagent (Invitrogen, MA, USA) and reverse-transcribed with a PrimeScript RT Reagent Kit. The resulting complementary DNA (cDNA) was used for qRT‒PCR with SYBR Green. The results were analysed with the delta-delta CT method, and values were normalized against GAPDH. The qRT‒PCR primers are shown in Supplementary Table S1.

### Western blot assay and antibodies

Cell lysates were prepared in radioimmunoprecipitation assay buffer, and proteins were resolved by SDS‒PAGE using sodium dodecyl sulfate–polyacrylamide gels followed by protein transfer onto nitrocellulose membranes (Sigma‒Aldrich, St. Louis, MO, USA). The gels were visualized by autoradiography. A GAPDH antibody served as the internal control. Other antibodies used for the western blot (WB) assay were as follows: GAPDH (Abclonal, AC001); B4GALT1 (Abcam, ab121326; SantaCruz, sc-515551); Cyclin D1 (Abclonal, A19038); CDK4 (Abclonal, A11136); PD-L1 (Cell Signaling Technology, 15,165; Abcam, ab213524; Abcam, ab205921; SantaCruz, sc-293425); TAZ (Cell Signaling Technology, 8418); DDDDK-Tag (Abclonal, AE063); and HA-tag (Abclonal, AE008).

### Plasmid DNA and siRNA transfection

*B4GALT1* cDNA was cloned into the expression vector pcDNA3.1. The X-tremeGEN™ HP DNA transfection reagent (Roche, Basel, Switzerland) was used for plasmid transfection, and the Lipo2000 reagent (Invitrogen, Shanghai, China) was used for small interfering RNA (siRNA) transfection, both performed according to the manufacturer’s instructions. In general, A549 or PC9 cells were seeded on coverslips in six-well plates and transfected with plasmid or siRNA on the second day. Cells were harvested 48 h after transfection for qRT‒PCR and western blot assays. The sequences of the siRNAs are summarized in Supplementary Table S1.

### MTT proliferation assays

Cell viability was measured with an MTT kit (Roche Applied Science, Indianapolis, IN, USA). The cells were seeded in 96-well plates at 2*10^3^/well posttransfection. Then, every 24 h, the cells were incubated in an incubator with 20 μl MTT for 4 h, and the crystals were dissolved at room temperature for 10 min with 150 μl dimethyl sulfoxide. The optical density was measured at 490 nm using a spectrophotometer (Thermo Fisher Scientific, Waltham, MA, USA).

### Colony formation assays

Five thousand cells were seeded in each well of a 6-well plate for the colony formation experiment, and growth medium was added as usual and replaced 1 week later. Methanol was applied for 15 min after the colonies had grown for 2 weeks, followed by 0.1% crystal violet (Sigma) staining for 30 min. Then, the formed clones were counted to reflect the colony-forming ability of the clones.

### Ethynyl deoxyuridine (EDU) analysis

EdU labelling and staining were performed with an EdU cell proliferation detection kit (RiboBio, Guangzhou, China). After adding cells at 5 × 10^3^ cells/well to 96-well plates, 50 mΜ EdU labelling medium was added to 96-well plates 48 h following transfection and incubated for 2 h in an incubator at 37 °C with 5% CO_2_. The cells were then treated with 4% paraformaldehyde and 0.5% Triton X-100 for anti-Edu working solution staining. The nuclei were labelled with diamidino-2-phenylindole. Fluorescence microscopy was performed to calculate the percentage of EdU-positive cells.

### Cell migration and invasion assays

Invasion and migration assays were carried out using Corning’s Transwell system (24 wells, 8 mm pore size, New York, NY, USA). For migration assays, 5 × 10^4^ cells posttransfection were seeded into the upper chambers of the plates with 350 μl of serum-free medium, and 700 μl of medium containing 10% FBS was added to the lower chambers. In Matrigel invasion assays, Matrigel (Sigma‒Aldrich)-precoated Transwell membranes were used. After incubation for 16 h, the upper surface cells were removed, and the cells that penetrated the membrane to the lower surface were stained with methanol and 0.1% crystal violet. Photographs were taken with an inverted microscope (Olympus, Tokyo, Japan).

### Wound closure assays

Cells were seeded onto a 6-well plate and cultured until they reached 90–100% confluence. Using a small pipette tip, confluent cells were scratched and washed twice with phosphate-buffered saline (PBS). Images of the same positions of each well at 0 or 16 h were taken with a microscope (Olympus, Tokyo, Japan). Wound closure was determined as a percentage of wound confluence using ImageJ software.

### Flow cytometric analysis of apoptosis and the cell cycle

After 48 h of transfection, trypsinized cells were collected and stained with fluorescein isothiocyanate (FITC)-Annexin V and propidium iodide using a FITC Annexin V Apoptosis Detection Kit (BD Biosciences, Franklin Lakes, NJ, USA). Live, dead, early apoptotic cells, and apoptotic cells were detected using a flow cytometer (FACScan; BD Biosciences) equipped with Cell Quest software (BD Biosciences). The proportion of apoptotic cells was calculated as the sum of the ratio of early apoptotic cells to late apoptotic cells. Cell cycle analysis was performed using the CycleTEST PLUS DNA Reagent Kit (BD Biosciences), and the results were analysed with FACScan. The percentages of cells in the G0-G1, S, and G2-M phases were determined and compared.

### CD8 + T-cell-mediated tumour cell-killing assays

Using Ficoll separation, we isolated peripheral blood mononuclear cells (PBMCs) from healthy donors’ peripheral blood. We also isolated splenocytes from the spleens of C57BL/6 mice. Positive selection of CD8 + T cells from PBMCs and splenocytes was performed using a CD8 MicroBeads Kit (Miltenyi Biotec 130–045-201, 130–116-478; Cologne, Germany). The isolated CD8 + T cells were cultured in RPMI 1640 medium supplemented with 10% foetal bovine serum, 1% antibiotics, 1X MEM nonessential amino acid (Gibco 11,140,050; Carlsbad, CA, USA), 1 mM sodium pyruvate (Gibco 11,360,070), 10 mM 4-(2-hydroxyethyl)-1-piperazineethanesulfonic acid (HEPES) (Gibco 15,630,080), 20 ng/ml IL2 (Peprotech 200–02; Cranbury, NJ, USA), and CD3/CD28 T-cell activator (Gibco 11161D) for 2 weeks. After transfection with siRNA or plasmid for 24 h, tumour cells and CD8 + T cells were cocultured at a 3:1 ratio (T cells: tumour cells) for 48 h. After removing T cells and cancer cell debris, crystal violet-stained living cancer cells were measured at 570 nm.

### Animal experiments

Five-week-old male BALB/c nude mice and C57BL/6 mice were purchased from Jiangsu Gempharmatech, Jiangsu, China. For subcutaneous xenograft experiments in BALB/c nude mice, 1 × 10^7^ A549 cells (si-*B4galt1* or si-control) were resuspended in 100 μl PBS and inoculated into the left and right flanks of mice. For subcutaneous xenograft experiments in C57BL/6 mice, 2 × 10^6^ LLC cells (si-*B4galt1* or si-control) suspended in 100 μl PBS were injected into the flanks. Once tumours reached approximately 100 mm^3^, mice were treated with mouse programmed cell death protein-1 (PD-1) mAb (BioXcell, BE0146; New Haven, CT, USA) or IgG isotype control (BioXcell, BE0089) by intraperitoneal injection (100 mg per mouse in 100 ml D-PBS buffer). Tumour sizes were measured every 2–3 days. Tumour weight was recorded, and volumes were estimated according to 1/2 × (length × width^2^).

### Flow cytometry analysis of immune cells

After C57BL/6 mice were sacrificed, fresh tumour tissue was excised and digested with collagenase IV (Gibco 17,104,019) and DNase I (Roche 10,104,159,001) for 2 h and filtered through 70-μm cell strainers to obtain a single-cell suspension. Cells were washed and then resuspended in 40% Percoll and centrifuged against 70% Percoll to sort lymphocytes. The cells were stimulated for 4 h with a leukocyte activation cocktail (BD Pharmingen, 550,583; San Diego, CA, USA) for intracellular cytokine staining. After blocking nonspecific antibody binding with CD16/CD32 antibody (553,141), surface staining was performed with the following antibodies: anti-CD45 (553,080), anti-CD3e (553,064), and anti-CD8α (551,162) mAbs from BD Pharmingen. After fixation and permeabilization with a Fixation/Permeabilization kit (BD Biosciences, 554,714), intracellular GZMB was stained with an anti-GZMB antibody (Biolegend, 372,204; San Diego, CA, USA). Stained cells were analysed by BD FACS Canto II. The data were analysed with FlowJo software.

### Cycloheximide (CHX) chase experiment

ells were then treated with 50 μg/ml cycloheximide (Selleck, Houston, TX, USA), and samples were collected at 0, 4, 8, and 12 h. Then, the samples were lysed to harvest proteins for subsequent western blot analysis.

### Immunohistochemical (IHC) and immunofluorescence assays

Tissue sections were obtained from the Pathology Department of Jiangsu Provincial People’s Hospital and were made from formalin-fixed paraffin-embedded samples from four MPLC patients. Sections were first stained with primary antibodies, followed by biotin-conjugated secondary antibodies and avidin–biotin-peroxidase complexes. Diaminobenzidine was used to visualize the target proteins. The images were analysed using 3D HISTECH QuantCenter 2.1 software. The H-score was used to quantify the immunohistochemical results. H-scores were used to quantify the immunohistochemical results, which were calculated according to the following formula: H-Score = ∑(pi × i) = (percentage of weak intensity × 1) + (percentage of moderate intensity × 2) + (percentage of strong intensity × 3) [22].

For multiple immunofluorescence, paraffin sections were dewaxed in water and then placed in a box filled with ethylenediaminetetraacetic acid (EDTA) antigen repair buffer (pH 8.0) in a microwave oven for antigen repair. After blocking with 3% bovine serum albumin (BSA) at room temperature for 30 min, the first primary antibody was added and incubated at 4 °C overnight, after which the slides were placed in PBS (pH 7.4) and washed by shaking on a decolorization shaker, followed by incubation with the corresponding secondary antibody at room temperature for 50 min. DAPI staining solution was applied to counterstain the nuclei with incubation for 10 min in the dark at room temperature. Finally, the slides were sealed with an anti-fluorescence quench sealer and placed under a fluorescence microscope for imaging. The antibodies employed for immunohistochemical and immunofluorescence were as follows: B4GALT1 (Abcam, ab32137); PD-L1 (Servicebio, GB11339A; Proteintech, 2B11D11); GZMB (Servicebio, GB14092); CD3 (Servicebio, GB11014); and CD8 (Servicebio, GB12068).

### Coimmunoprecipitation assays

Following the manufacturer’s instructions, we performed coimmunoprecipitation experiments using the Pierce Classic Magnetic IP/Co-IP Kit (Thermo Fisher Scientific, San Jose, CA). Briefly, A549 cells were transfected with B4GALT1-HA (or empty vector) and PD-L1-Flag/TAZ-Flag (or empty vector) for 24 h, and then cell lysates were incubated with DDDDK-Tag mAb (Abclonal, AE063) or anti-HA-Tag mAb (Abclonal, AE008) at room temperature for 2 h. Then, Protein A/G magnetic beads were bound to the antigen/antibody complexes at 4 °C overnight, followed by two washes with lysis/wash buffer and one wash with purified water. Finally, the beads were resuspended in 100 μl of Lane Marker Sample Buffer and boiled for 10 min for subsequent western blotting.

### GST pull-down assay

GST and GST–B4GALT1 proteins were purchased from OriGene (WX0383AA, WX02BF90), and we performed GST pull-down assays using the Pierce™ GST Protein Interaction Pull-Down Kit (Thermo Fisher Scientific, San Jose, CA). Briefly, we immobilized 150 µg GST and GST-B4GALT1 fusion proteins in 50 µL glutathione agarose and incubated them together for 60 min at 4 °C with gentle rocking. Then, A549 cell lysates were added to the immobilized GST-B4GALT1 and GST. The two fusion proteins were incubated overnight at 4 °C with gentle rocking. The bound proteins were eluted with 10 mM glutathione elution buffer and examined by SDS‒PAGE and western blotting.

### Dual-luciferase reporter assays

Following seeding in a 24-well plate, cells were cotransfected with the *CD274* promoter-reporter gene vector, the Renilla luciferase vector, and either *B4GALT1* pcDNA or *TAZ* siRNA. After 48 h of transfection, cell lysates were prepared using the Dual-Luciferase Reporter Assay System (Promega, Madison, WI, USA). Renilla luciferase activity was used as the internal control for normalizing the luciferase activity.

### Statistical analysis

Statistical analyses were performed using IBM SPSS 24 software and the R 3.5.1 package for statistical computing. For comparisons of variables between two groups, two-tailed Student’s t tests and Wilcoxon tests were used, as appropriate. Linear relationships between two variables were evaluated with Pearson correlations. Disease-free survival (DFS) and overall survival (OS) were estimated by the Kaplan–Meier method, and comparisons were made by the log-rank test. Two-sided P values were used, and statistical significance was defined as *P* < 0.05.

## Results

### Identification of *B4GALT1* as a key factor in the tumorigenesis of early-stage LUAD

**Fig. 1:**
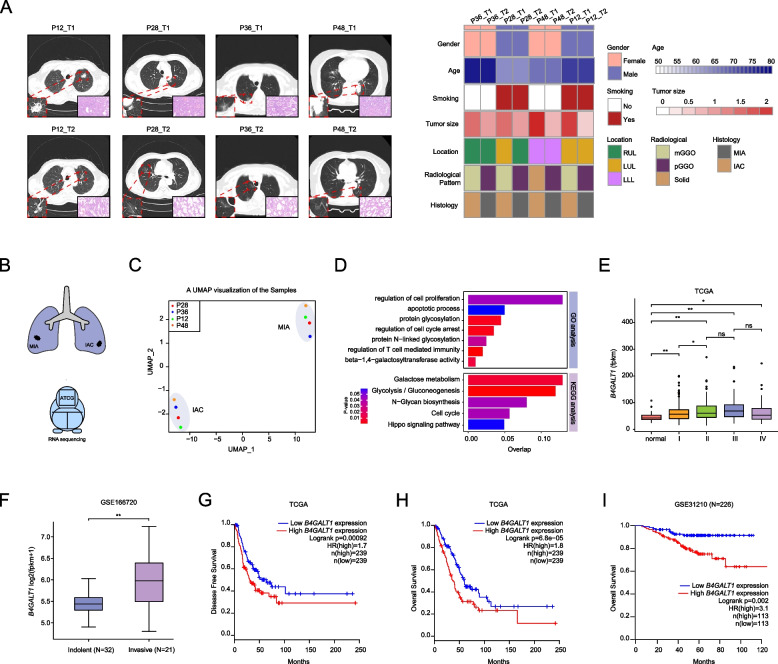
Identification of *B4GALT1* as a key factor in the tumorigenesis of early-stage LUAD. **A** The study cohort included four MPLC patients with MIA and IAC tumours. On the left is the pathological and corresponding radiological images of eight tumours. On the right is the heatmap indicating the clinical parameters. mGGO: mixed ground-glass opacity; pGGO: pure ground-glass opacity; RUL: right upper lobe; LUL: left upper lobe; LLL: left lower lobe. **B** Surgical specimens from these patients were processed for RNA sequencing. **C** Clustering of RNA-seq data using UMAP. **D** GO and KEGG pathway enrichment analyses of genes upregulated in IAC. **E ***B4GALT1* expression levels in LUAD grouped by disease stage (data retrieved from TCGA database). FPKM: fragments per kilobase of exon model per million mapped fragments. **F ***B4GALT1* expression was significantly increased in invasive LUAD compared with indolent LUAD (data were obtained from GSE166720). **G** Disease-free survival curves stratified by *B4GALT1* expression (data retrieved from TCGA database). **H** Overall survival curves stratified by *B4GALT1* expression (data retrieved from TCGA database). **I** Overall survival curves stratified by *B4GALT1* expression (data retrieved from GSE31210). *P < 0.05, **P < 0.01, n.s., not significant

Eight LUAD samples obtained from four MPLC patients were collected, and their radiomic features and clinicopathologic characteristics are summarized in Fig. 1A. In each patient, the two primary tumours were of different pathological types, MIA or IAC. Then, we performed RNA sequencing to explore key molecular events driving the progression of preinvasive LUAD to invasive LUAD in these samples (Fig. 1B). Subsequently, unsupervised clustering of RNA-sequencing data with UMAP identified two clusters that corresponded exactly to the MIA and IAC lesions of these four patients (Fig. 1C), suggesting distinct transcriptional characteristics for MIA and IAC. GO and KEGG analyses revealed that genes highly expressed in IAC were significantly enriched in glucose-metabolic pathways (Fig. 1D). Furthermore, N-glycan biosynthesis, with enrichment of beta-1,4-galactosyltransferase activity signalling pathways, was also found, indicating that glycosylation played a key role in the development of early-stage lung cancer. As one type of glycosylation, the N-glycosylation pattern can regulate protein stability, and beta-1,4-galactosyltransferase is a key enzyme involved in N-glycan biosynthesis. Here, we focused on *B4GALT1*, a gene encoding β-1,4-galactosyltransferase, which was the most significantly upregulated gene in IAC samples. Therefore, we speculated that *B4GALT1* may play a key role in the evolution of MIA to IAC for early-stage LUAD. Further analysis of TCGA-LUAD data showed that *B4GALT1* mRNA expressions were indeed increased with stage in early-stage LUAD but not in advanced LUAD (Fig. 1E). Moreover, the GSE166720 dataset, which classified 53 early-stage lung adenocarcinomas into indolent (AIS and MIA) and invasive tumours (IAC) according to pathological grade, showed that *B4GALT1* mRNA expressions were higher in invasive tumours than in indolent tumours (Fig. 1F). Additionally, high *B4GALT1* expression predicted worse DFS and OS in TCGA-LUAD (Fig. 1G-H). High *B4GALT1* also predicted poor OS in the GSE31210 dataset with stage I and stage II LUAD patients (Fig. 1I). Together, the results suggested that *B4GALT1* might play a crucial role in the tumorigenesis of early-stage LUAD.

### B4GALT1 regulates LUAD cell proliferation and metastasis in vitro and in vivo

**Fig. 2:**
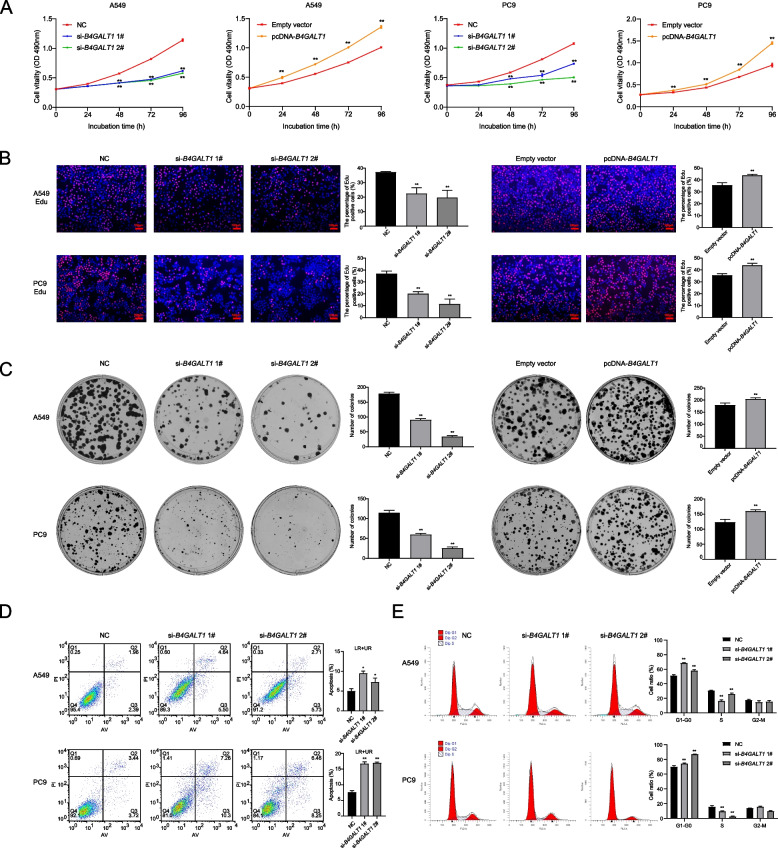
*B4GALT1* regulates LUAD cell proliferation in vitro and in vivo. **A** MTT assays were used to determine the viability of A549 and PC9 cells transfected with si-*B4GALT1* or pcDNA-*B4GALT1*. **B** EdU assays were used to analyse the proliferation of A549 and PC9 cells after transfection. **C** Colony formation assays were performed to detect the clonogenicity of A549 and PC9 cells. **D** The apoptotic rates of transfected A549 and PC9 cells were analysed using flow cytometry. LR represents early apoptotic cells. UR represents terminal apoptotic cells. **E** The cell cycles of A549 and PC9 cells were determined by flow cytometry. *P < 0.05, **P < 0.01

To explore the functional role of *B4GALT1* in the tumorigenesis of LUAD, we used a specific siRNA and an overexpression plasmid to manipulate the expression of *B4GALT1* in A549 and PC9 cells (Figure S1A-C). MTT tests, colony formation assays, and EdU experiments were performed to identify the role of *B4GALT1* in cell proliferation. MTT tests showed that *B4GALT1* knockdown could significantly decrease cell viability. In contrast, *B4GALT1* overexpression increased cell viability (Fig. 2A). Similarly, EdU assays revealed that *B4GALT1* had a significant impact on LUAD cell proliferation (Fig. 2B). Colony formation assays demonstrated that *B4GALT1* expression also had an impact on clonogenic ability (Fig. 2C). Moreover, *B4GALT1* knockdown significantly induced LUAD cell apoptosis (Fig. 2D). Then, we determined whether *B4GALT1* affected LUAD cell proliferation by altering the cell cycle. Flow cytometry results showed that *B4GALT1* knockdown was accompanied by cell cycle arrest at the G1/G0 stage (Fig. 2E). We then examined the expression of G1/S phase transition-related proteins. As determined by western blotting, *B4GALT1* knockdown or overexpression led to decreases or increases in cyclin D1 and CDK4 protein levels in LUAD cell lines (Figure S1D).

**Fig. 3:**
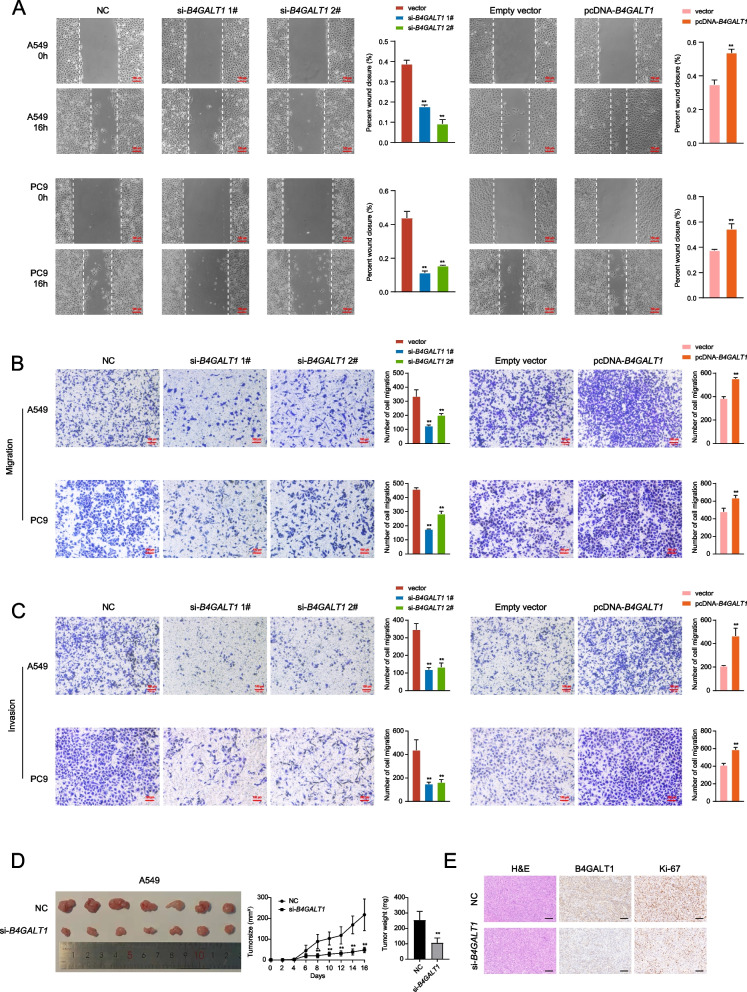
*B4GALT1* regulates LUAD cell migration and invasion. **A** Wound healing assays were used to analyse the migratory activity of A549 and PC9 cells transfected with si-*B4GALT1* or pcDNA-*B4GALT1*. **B** Transwell assays were used to detect the migration ability of transfected A549 and PC9 cells. **C** Transwell chambers coated with Matrigel were used to assess invasion after transfection with si-*B4GALT1* or pcDNA-*B4GALT1* compared with the control groups in A549 and PC9 cells. **D** Tumours were established in BALB/c nude mice by subcutaneous injection of si-B4GALT1-transfected or control A549 cells. Tumour volumes were calculated after injection every 2 days. The tumour growth curves are shown in the left panel. Tumour weight was assessed from excised tumours (right panel). **E** Representative images of tumours subjected to H&E staining and IHC staining (B4GALT1, Ki-67). Scale bars, 100 µm. *P < 0.05, **P < 0.01

Next, we tested the potential impact of *B4GALT1* on cell migration and invasion. In wound-healing assays, *B4GALT1* expression significantly impaired wound closure (Fig. 3A). Transwell assays also demonstrated that *B4GALT1* knockdown impeded tumour migration. Conversely, *B4GALT1* overexpression significantly promoted cell migration (Fig. 3B). Furthermore, *B4GALT1* knockdown restrained LUAD cells from invading through matrices, whereas *B4GALT1* overexpression facilitated the invasion of LUAD cells (Fig. 3C).

We subcutaneously injected A549 cells into BALB/c nude mice to determine whether *B4GALT1* influences LUAD tumour growth in vivo. The tumour volume in the *B4GALT1* knockdown group decreased significantly compared with that in the control group. At the experimental endpoint, the average weight of tumours in the *B4GALT1* knockdown group was significantly lower than that in the control group (Fig. 3D). Moreover, Ki67 staining showed a decrease in the proportion of Ki67-positive cells among tumours formed by *B4GALT1* knockdown (Fig. 3E).

### *B4GALT1* limited CD8 + T-cell infiltration and activity

**Fig. 4:**
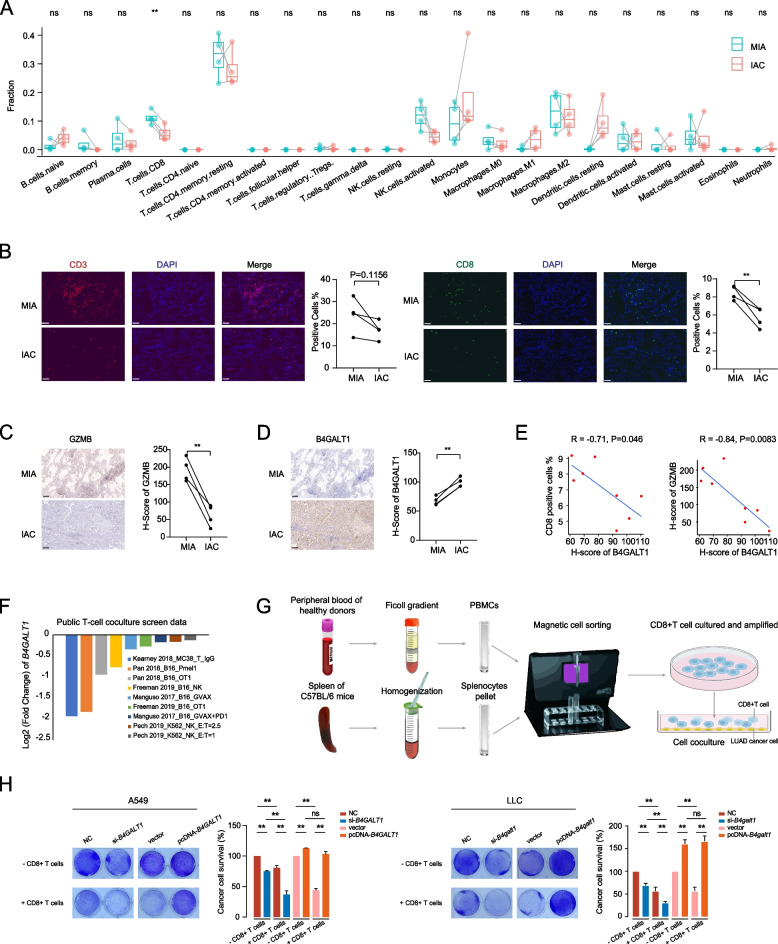
*B4GALT1* limited CD8 + T-cell infiltration and activity. **A** Differences in immune composition between the MIA and IAC groups were determined using CIBERSORT. **B** Representative immunofluorescence staining images of CD3 and CD8 in MIA and IAC tumours. Scale bars, 50 µm. Quantifications are on the right. **C** Representative immunohistochemical images (left panel) and comparison of H-scores of GZMB in MIA and IAC tumours (right panel). Scale bars, 200 μm. **D** Representative immunohistochemical images (left panel) and comparison of H-scores of B4GALT1 in MIA and IAC tumours (right panel). Scale bars, 200 μm. **E** The linear correlations between the H-scores of B4GALT1 and the percentages of CD3, CD8, and H-scores of GAMB in eight tumours from four MPLC. **F** Log2-fold changes of *B4GALT1* expression in the public T-cell coculture screen data. **G** Illustration showing the in vitro procedure of human and mouse CD8 + T-cell isolation and cocultivation with cancer cells. **H** LUAD cells cocultured with activated CD8 + T cells for 48 h were subjected to crystal violet staining and quantified at 570 nm. The ratio of LUAD cells to T cells was 1:3. **P < 0.01, n.s., not significant

Through GO analysis of highly expressed genes in IAC, we also detected an enrichment of genes with functions associated with the T-cell immune response (Fig. 1D). Previous studies have reported increased immunosuppression during the progression of LUAD from MIA to IAC [10]. Therefore, we applied the CIBERSORT algorithm to estimate the relative abundance of immune cells among MIA and IAC tumours. We found that IAC had less CD8 + T-cell infiltration than MIA (Fig. 4A). Subsequent immunofluorescence staining analysis also showed fewer CD8 + T cells in IAC tumours than in MIA tumours (Fig. 4B). Immunohistochemical staining for GZMB, the key CD8 + T-cell effector molecule, revealed that IAC tumors had lower GZMB level, indicating the attenuated activity of CD8 + T cells in IAC tumours (Fig. 4C). To determine whether *B4GALT1* induced this alteration in the immune status, immunohistochemical staining for BGALT1 was carried out on these samples (Fig. 4D), and a clear inverse correlation between B4GALT1 level and the number of CD8 + T cells was found, as well as for GZMB (Fig. 4E). We further validated these correlations in the TCGA-LUAD dataset. *B4GALT1* mRNA levels negatively correlated with CD8 + T-cell infiltration (Figure S2A), and tumours with higher *B4GALT1* mRNA levels had higher tumour immune dysfunction and exclusion (TIDE) scores than those with lower *B4GALT1* expression, suggesting the immunosuppressive role of *B4GALT1* in LUAD tumorigenesis (Figure S2B). In addition, the correlation between the cytotoxic T-lymphocyte (CTL) level and OS for LUAD patients was assessed under the condition of high or low *B4GALT1* mRNA levels in two independent GEO datasets. As shown in Figure S2C, high expression of *B4GALT1* was inversely related to a CTL-mediated survival benefit, implying that in tumour microenvironments characterized by high *B4GALT1* expression, CTLs may become dysfunctional.

To determine whether *B4GALT1* was an important regulator of dysfunctional T cells, we explored data from publicly available clustered regularly interspersed short palindromic repeats (CRISPR) screens in the context of murine cancer cells cocultured with murine primary T cells that specifically target cancer cell antigens. Based on nine such screens in five independent studies [23–26], we found that gRNAs targeting *B4GALT1* were consistently negatively selected (Fig. 4F). These results indicated that *B4GALT1* could contribute to CD8 + T-cell dysfunction and may make LUAD cells more susceptible to CD8 + T-cell-mediated cytotoxicity when inactivated. To further verify this conclusion, T-cell-mediated tumour cell-killing assays were performed to test the impact of *B4GALT1* expression on CD8 + T-cell activity, which were isolated from the peripheral blood of healthy donors and the spleens of C57BL/6 mice (Fig. 4G). As expected, *B4GALT1* inhibition significantly enhanced LUAD cell death mediated by CD8 + T cells, and *B4GALT1* upregulation rendered LUAD cells more resistant to CD8 + T cells in both the A549 and LLC cell lines (Fig. 4H).

### B4GALT1 interacts with PD-L1 and enhances PD-L1 protein stability by mediating its glycosylation, thus promoting immune escape in LUAD

**Fig. 5:**
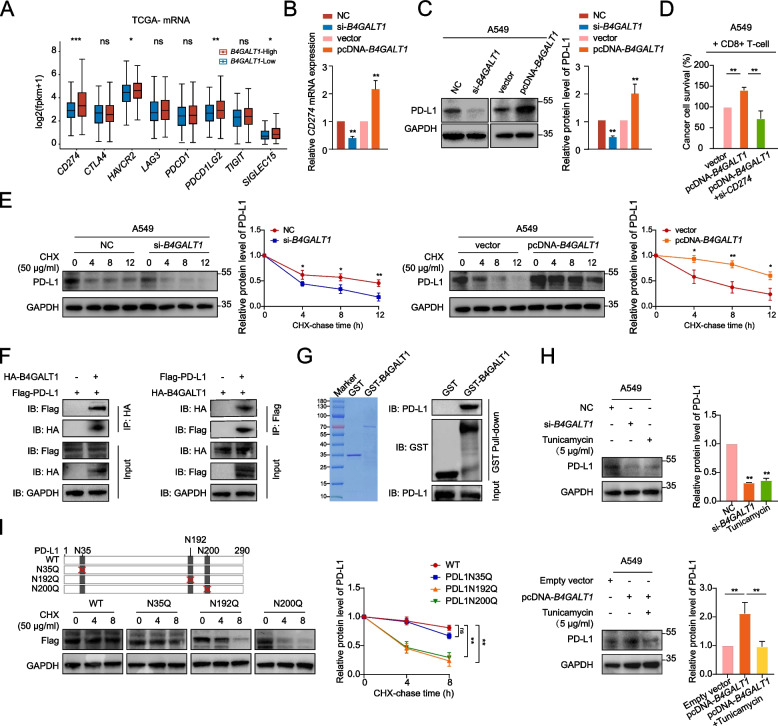
B4GALT1 interacts with PD-L1 and enhances PD-L1 protein stability by mediating its glycosylation, thus promoting immune escape in early-stage LUAD. **A** The differential expression of immune checkpoint proteins in tumours with high and low *B4GALT1* expression (data retrieved from TCGA-mRNA database). **B**, **C** A549 cells were transfected with si-*B4GALT1* or pcDNA-*B4GALT1*, and PD-L1 expression was analysed by qRT‒PCR and western blotting. **D** A549 cells cocultured with activated CD8 + T cells for 48 h were subjected to crystal violet staining and quantified at 570 nm. The ratio of A549 cells to T cells was 1:3. **E** Western blot analysis of PD-L1 expression in A549 cells with or without B4GALT1 knockdown or overexpression followed by treatment with CHX. **F** Coimmunoprecipitation experiments indicated that B4GALT1 interacted with PD-L1 in both A549 cell lines. **G** Coomassie staining of GST and GST-B4GALT1 proteins (left). Detection of PD-L1 bound to GST-B4GALT1 or GST in a GST pull-down assay (right). **H** Western blot analysis of PD-L1 levels in *B4GALT1* knockdown cells, *B4GALT1*-overexpressing cells, and tunicamycin (TM)-treated cells. **I** Western blot analysis of Flag-tagged WT or mutated PD-L1 expression levels in *B4GALT1*-overexpressing A549 cells followed by treatment with CHX. *P < 0.05, **P < 0.01, n.s., not significant

Increasing evidence indicates that immune suppression is linked to the dysregulation of immune checkpoints [27, 28]. Therefore, we compared the expression levels of well-known immune checkpoint molecules between tumours with high and low *B4GALT1* expression (Fig. 5A). Notably, *CD274* (PD-L1) exhibited the most significantly higher expression in *B4GALT1*-high tumours than in *B4GALT1*-low tumours. CCLE and TCGA data further substantiated that high *B4GALT1* expressers had higher expression levels of PD-L1 mRNA and protein (Figure S3A-B). Furthermore, there was a highly significant positive correlation between B4GALT1 and PD-L1 level among our MPLC samples (Figure S3C). In agreement with the results above, decreased or increased mRNA and protein levels of PD-L1 were elicited by *B4GALT1* knockdown or overexpression in A549 and LLC cells (Fig. 5B-C, Figure S3D-F). Therefore, it is reasonable to hypothesize that *B4GALT1* mediates CD8 + T-cell dysfunction by affecting PD-L1 expression in LUAD. This speculation was confirmed by the restoration of CD8 + T-cell killing ability by *CD274* knockdown in *B4GALT1*-overexpressing LUAD cells (Fig. 5D, Figure S3G).

Next, we asked how *B4GALT1* affects the level of PD-L1. *B4GALT1* is a key gene involved in N-glycan biosynthesis and participates in the formation of N-glycosylation. Therefore, we speculated that N-linked glycosylation may be involved in the *B4GALT1*-mediated regulation of PD-L1 via a mechanism involving posttranslational modification. First, after treatment with cycloheximide (CHX), we found that *B4GALT1* depletion reduced the half-life of the PD-L1 protein in both A549 and LLC cells, whereas stable *B4GALT1* expression significantly increased the protein stability of PD-L1 (Fig. 5E, Figure S3H). Besides, co-IP verified the interaction between B4GALT1 and PD-L1 (Fig. 5F). To confirm the interaction biochemically, the GST fusion protein containing B4GALT1 was expressed and purified to perform GST pulldown assays. The result showed that GST-B4GALT1 could pull down PD-L1 in A549 cell lysates (Fig. 5G). Then, we sought to investigate whether *B4GALT1* regulates the PD-L1 protein through N-linked glycosylation. A549 and LLC cells were treated with tunicamycin (an N-linked glycosylation inhibitor), and PD-L1 expression was significantly downregulated compared with that in nontreated cells, consistent with *B4GALT1* knockdown. Moreover, tunicamycin rescued the PD-L1 expression induced by *B4GALT1* overexpression (Fig. 5H, Figure S3I). To further identify the N-linked glycosylation site of PD-L1, we first employed the NetNGlyc 1.0 server tool to predict the putative N-linked glycosylation motifs of PD-L1. Then, *B4GALT1*-overexpressing cells were transfected with FLAG-tagged plasmids carrying either a full-length coding sequence of PD-L1 or a coding sequence containing a mutated N-linked glycosylation site at N35, N192, or N200 for CHX analysis (Fig. 5I). The results showed that mutating the N-linked glycosylation sites at N192 and N200 significantly reduced PD-L1 protein stability even in the presence of *B4GALT1* overexpression, suggesting that these residues are the N-linked glycosylation sites of PD-L1 modified by *B4GALT1*. Taken together, the above results suggested that B4GALT1 interacts with PD-L1 by mediating its glycosylation, thus promoting immune escape in LUAD.

### *B4GALT1* regulates TAZ glycosylation to promote PD-L1 transcription

**Fig. 6:**
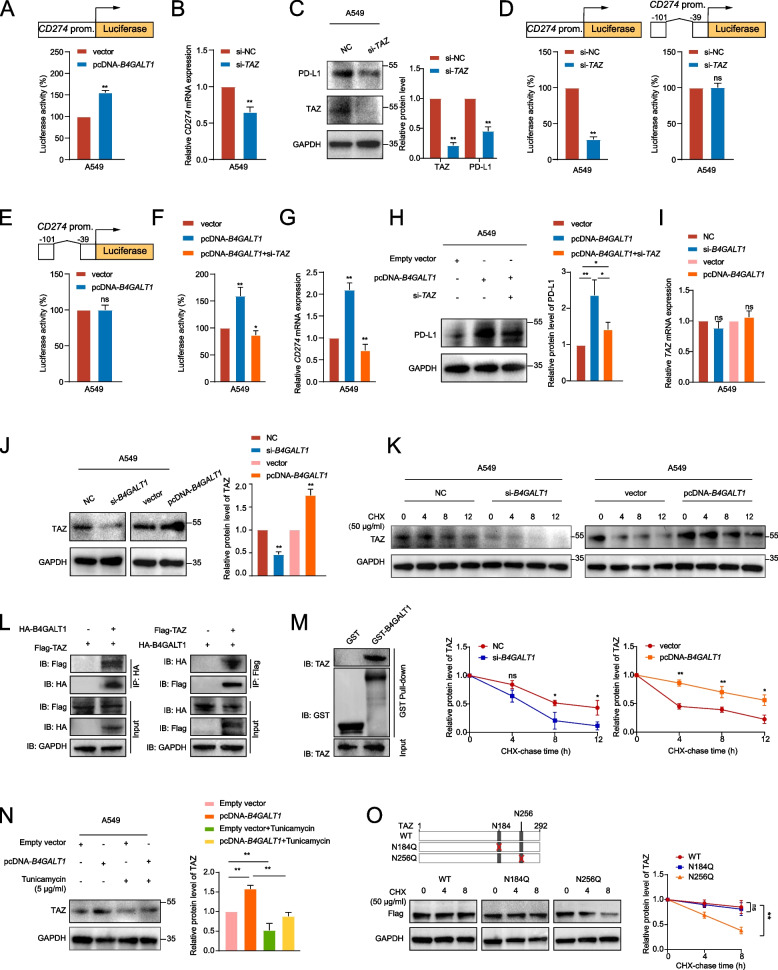
*B4GALT1* regulates TAZ glycosylation to promote PD-L1 transcription. **A** Analysis of luciferase activity in A549 cells transfected with a full-length *CD274* promoter and a *B4GALT1* overexpression plasmid. The results are expressed as the fold change compared with the control cells. **B**, **C** A549 cells with TAZ *knockdown* and PD-L1 expression were analysed by qRT‒PCR and western blotting. **D** Analysis of the luciferase activity of *CD274* in A549 cells transfected with a full-length *CD274* promoter construct or a construct with deletion of the -101 to -39 *CD274* promoter region and knockdown of TAZ. The results are expressed as fold changes compared with the control cells. **E** Analysis of the luciferase activity of *CD274* in A549 cells transfected with the *CD274* promoter construct with deletion of the -101 to -39 region and a *B4GALT1* overexpression plasmid. The results are expressed as fold changes compared with the control cells. **F** Analysis of the luciferase activity of *CD274* in A549 cells transfected with the full-length *CD274* promoter construct, along with *B4GALT1* pcDNA or *TAZ* siRNA. The results are expressed as fold changes compared with the vector cells. **G**, **H** qRT‒PCR and western blot analyses revealed that *TAZ* knockdown mitigated the *B4GALT1*-induced upregulation of PD-L1 mRNA and protein levels. **I**, **J** A549 cells were transfected with si-*B4GALT1* or pcDNA-*B4GALT1*, and TAZ expression was analysed by qRT‒PCR and western blotting. **K** Western blot analysis of TAZ expression in A549 cells with or without *B4GALT1* knockdown or overexpression followed by treatment with CHX. **L** Coimmunoprecipitation experiments indicated that B4GALT1 interacted with TAZ in A549 cells. **M** Detection of TAZ bound to GST-B4GALT1 or GST in a GST pull-down assay. **N** Western blot analysis of TAZ levels in *B4GALT1* knockdown cells, *B4GALT1*-overexpressing cells, and tunicamycin (TM)-treated cells. *P < 0.05, **P < 0.01, n.s., not significant. **O** Western blot analysis of Flag-tagged WT or mutated TAZ expression levels in *B4GALT1*-overexpressing A549 cells followed by treatment with CHX

Our data above demonstrated that *B4GALT1* could enhance PD-L1 protein stability. In addition, we noticed changes in *CD274* mRNA after *B4GALT1* knockdown or overexpression (Fig. 5B, Figure S3E). These results indicated that *B4GALT1* may also have a transcriptional effect on *CD274*. To test this hypothesis, luciferase reporter assays were performed and revealed that *CD274* promoter activity was enhanced by *B4GALT1* overexpression (Fig. 6A, Figure S4A). In addition, interestingly, we found that highly expressed genes in IAC tumours were enriched in the Hippo signalling pathway (Fig. 1D). In prior studies, WW domain-containing transcription regulator 1, (*TAZ*), an important transcription factor of the Hippo signalling pathway, was found to increase *CD274* promoter activity to induce PD-L1 upregulation [29, 30]. Here, we also validated the regulation of *CD274* by *TAZ* in LUAD. *TAZ* knockdown led to substantial decreases in *CD274* mRNA and protein expression levels in A549 and LLC cells (Fig. 6B-C, Figure S4B-C). Further reporter gene assays revealed that *TAZ* knockdown decreased the activity of the full-length *CD274* promoter but not the promoter construct with a deleted region (Fig. 6D, Figure S4D). Importantly, *B4GALT1* overexpression did not affect the activity of the *CD274* promoter construct with the deleted region, indicating that *B4GALT1* affects *CD274* promoter activity through *TAZ* (Fig. 6E). Furthermore, *TAZ* knockdown significantly mitigated the *B4GALT1*-induced increases in full-length *CD274* promoter activity and *CD274* mRNA levels (Fig. 6F-G, Figure S4E-F), suggesting that *B4GALT1* mediates the transcriptional regulation of *CD274* through *TAZ*. Moreover, *TAZ* knockdown reversed *B4GALT1*-induced PD-L1 protein level (Fig. 6H, Figure S4G).

We further sought to explore the mechanism by which *B4GALT1* impacts TAZ level. Through the analysis of TCGA and GEO datasets, we observed that *B4GALT1* expression was significantly related to TAZ level at the protein level instead of the mRNA level (Figure S5A-B), consistent with the qRT‒PCR and WB results (Fig. 6I-J, Figure S5C-D). Thus, we suspected that TAZ protein may be regulated by *B4GALT1* at the posttranslational level by acquiring N-glycan modifications. Through CHX analysis, we found that *B4GALT1* could increase TAZ protein stability (Fig. 6K, Figure S5E). Moreover, co-IP (Fig. 6L) and GST pull-down assays (Fig. 6M) suggested that B4GALT1 directly interacted with TAZ. Furthermore, TAZ protein levels were significantly decreased after tunicamycin treatment in *B4GALT1*-overexpressing cells (Fig. 6N, Figure S5F), indicating that the N-linked glycosylation of TAZ is regulated by *B4GALT1*. Then, we applied the NetNGlyc 1.0 server tool to predict the potential N-linked glycosylation sites of TAZ and mutated the N184 and N256 sites (Fig. 6O). The results of CHX analysis indicated that *B4GALT1* overexpression could not promote TAZ protein stability when the N-linked glycosylation site at N256 was mutated, implying that N256 is the N-linked glycosylation site of TAZ modified by *B4GALT1*.

These results demonstrated the translational modification of PD-L1 which was mediated by *B4GALT1*/*TAZ*.

### *B4GALT1* inhibition augments the efficacy of anti-PD-1 therapy in LUAD

**Fig. 7:**
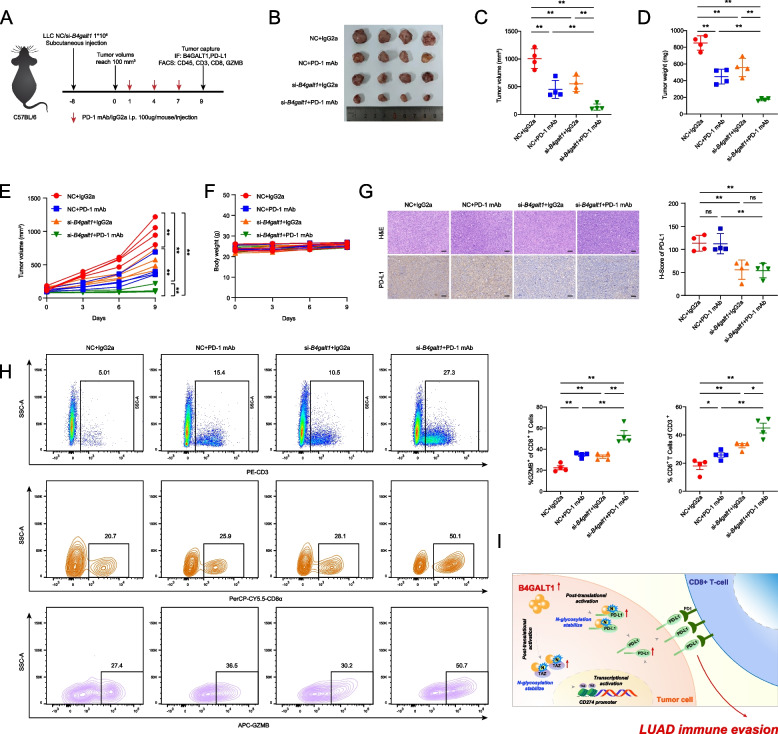
*B4GALT1* inhibition augments the efficacy of anti-PD-1 therapy in LUAD. **A** C57BL/6 mice were implanted with 2*10^6^ si-*B4galt1* or control LLC cells that received PD-1 mAb or IgG isotype control (IgG2a) treatment. The treatment plan is shown. **B** Images of subcutaneous xenograft tumours harvested after euthanizing C57BL/6 mice. **C**, **D** Summary of volume and weight data of tumours. **E**, **F** Plots of tumour volumes and mouse body weights measured every 3 days. **G** Representative immunohistochemical images (left panel) and comparison of H-scores of PD-L1 in tumours (right panel). Scale bars, 50 μm. **H** Fluorescence-activated cell sorting of CD3 + in CD45 + cells, CD8 + in CD3 + T cells, and granzyme B in CD8 + T cells from xenografts and quantification. **I** Schematic model illustrating the mechanism of *B4GALT1*-mediated tumorigenesis and immune escape in LUAD. The red plus signs represent activation. *P < 0.05, **P < 0.01, n.s., not significant

To evaluate the immune regulatory effect of *B4GALT1 *in vivo, we utilized the LLC mouse LUAD cell line to construct subcutaneous xenografts in immunocompetent C57BL/6 mice, which were then treated with the PD-1 mAb or IgG isotype CTRL (IgG2a) (Fig. 7A). We found that *B4galt1* knockdown limited tumour growth compared to the control group, consistent with PD-1 mAb treatment. Moreover, the *B4galt1* inhibition and PD-1 blockade combination therapy group showed the most significant reduction in tumour burden compared with the other groups, and neither body weight loss nor other common toxic effects were observed (Fig. 7B-F). Tumour samples were harvested for further analysis at the end of the treatment, and IHC staining and flow cytometry analysis showed that *B4galt1* deficiency significantly decreased PD-L1 level and increased CD8 + T-cell and CD8 + GZMB + cell densities (Fig. 7G-H). In particular, cotreatment with PD-1 mAb and *B4galt1* inhibition led to the most significant enrichment of activated CD8 + T cells in tumour regions (Fig. 7H). These results confirmed that *B4galt1* inhibition enhanced CD8 + T-cell infiltration and function by downregulating PD-L1 expression, thus improving the efficacy of anti-PD-1 therapy. Collectively, these results indicated that *B4GALT1* contributed to tumour immune escape by upregulating PD-L1 expression and inhibiting CD8 + T-cell infiltration.

## Discussion

In recent years, the detection rate of early lung cancer has increased rapidly and drastically due to the widespread availability of CT-guided screening [31, 32]. Early-stage lung cancer can be treated with minimally invasive surgery, which requires only a short hospital stay and is associated with a high cure rate and good tolerance. New ideas for treating early lung cancer can be derived from a better understanding of the molecular mechanisms involved. Host immune surveillance and cancer cells interact to shape cancer evolution [33]. It is well documented that even stage I lung cancer significantly compromises T-cell immunity, which indicates that these cancers have already begun escaping immune surveillance [34, 35]. In this work, we also aimed to elucidate the immune evasion of early-stage LUAD and its underlying molecular mechanisms.

There is evidence that glycosylation is altered in the pathogenesis of lung cancer [13]. As a result of this alteration, many proteins involved in tumour progression are activated. In this study, we first analysed RNA sequencing data from four pairs of MIA and IAC with the same genetic background based on MPLC models and found that IAC tumours significantly activated the N-glycan biosynthesis signalling pathway, among which *B4GALT1* was the most significantly differentially expressed gene. In previous studies, *B4GALT1* expression predicted prognosis in pancreatic ductal adenocarcinomas and bladder cancer [18, 19]. However, its role in lung cancer is still not clear. Our results further demonstrated that *B4GALT1* contributed to carcinogenesis via the augmentation of tumour cell proliferation, migration, and invasiveness in LUAD.

In addition, *B4GALT1* was identified to play a pivotal role in immune evasion to participate in the malignant behaviours of LUAD tumour cells. We found that *B4GALT1* was negatively correlated with the proportion of CD8 + T cells and was associated with T-cell dysfunction and further found that *B4GALT1* was positively correlated with PD-L1 level, thereby mediating immune escape.

Overexpressing PD-L1 in tumours results in an inhibitory signal that promotes T-cell exhaustion, allowing tumours to escape the immune system [36, 37]. There are multiple processes involved in PD-L1 regulation, such as transcriptional upregulation by MYC, HIF1/2α, NF-κB, MAPK, PTEN/PI3K, and EGFR, posttranscriptional regulation by miRNAs, and posttranslational modification by N-glycosylation, phosphorylation, ubiquitination, and palmitoylation [38]. Several studies have identified that glycosylation could regulate PD-L1 and suppress PD-L1 protein degradation, contributing to T-cell immunosuppression [39, 40]. Here, we first provided evidence that at the posttranscriptional level, B4GALT1 interacted with PD-L1 and stabilized the PD-L1 protein via N-linked glycosylation in LUAD. Moreover, we showed that *B4GALT1* expression can regulate the immune escape ability and T-cell killing effect of LUAD.

We also noted that *B4GALT1* had a significant impact on *CD274* mRNA levels, indicating that PD-L1 could be regulated by *B4GALT1* at the transcriptional level. We observed that IAC tumours activated the Hippo signalling pathway. Studies have found that *TAZ*, the key effector of the Hippo signalling pathway, interacted with the transcription factor *TEAD* to increase *CD274* promoter activity, which was sufficient to restrain T-cell function [29, 30]. Then, we further proved that *B4GALT1* stabilized the TAZ protein via glycosylation, which in turn facilitated *CD274* transcription. In our animal studies, we showed that *B4GALT1* inhibition significantly decreased the tumour volume, which was similar to the effect of PD-1 blockade. Additionally, we proposed an effective strategy for boosting antitumour immunity of anti-PD-1 therapy through *B4GALT1* inhibition.

## Conclusions

Taken together, based on unique MPLC models, we revealed that *B4GALT1* promoted the immune evasion and tumorigenesis of early-stage LUAD and identified a novel mechanism whereby *B4GALT1* regulated PD-L1 at the transcriptional level by stabilizing TAZ protein and at the posttranslational level directly via N-linked glycosylation protein modification of PD-L1. This study provides a certain theoretical basis for applying *B4GALT1* as a potential therapeutic target for LUAD (Fig. 7I).

## Supplementary Information

**Additional file 1: Figure S1. **(A-B) qRT-PCR was performed to detect the *B4GALT1* mRNA expression after siRNA-mediated knockdown and plasmid-mediated overexpression in A549 and PC9 cells. (C) Western blot was performed to detect the B4GALT1 protein expression. (D) The protein expression levels of CDK4 and Cyclin D1 in control and ectopic *B4GALT1*-expressing A549 and PC9 cells were determined using western blotting analyses. * P < 0.05, ** P < 0.01. **Figure S2. **(A) The linear correlations between the *B4GALT1* expression and the CD8+ T-cell expression in the TCGA-LUAD dataset. (B) Comparison of TIDE scores between the *B4GALT1*-high group and *B4GALT1*-low group. (C) Comparison of overall survival between CTL top (samples above average CTL values among all samples) and CTL bottom (samples below average CTL values among all samples) groups in patients with different *B4GALT1* levels. The two-sided Wald test in the Cox-PH regression was applied to compute the association between the CTL level and overall survival, and samples were split by the best separation strategy according to the *B4GALT1 *expression coefficients in the Cox-PH regression model. ** P < 0.01.** Figure S3. **(A-B) The differential expression of *CD274* in tumours with high expression of *B4GALT1* and low expression of *B4GALT1* in the CCLE mRNA dataset (A) and the differential expression of PD-L1 in tumours with high expression of *B4GALT1* and low expression of *B4GALT1 *in the TCGA-protein dataset (B). RPKM: reads per kilobase per million mapped reads; RPPA: reverse phase protein array. (C) Representative immunohistochemical images (left panel) and the association between B4GALT1 and PD-L1 level in MPLC tumours (right panel). Scale bars, 200 μm. (D) qRT-PCR was performed to detect the *B4galt1* mRNA expression after siRNA-mediated knockdown in LLC cells. (E-F) LLC cells were transfected with si-*B4galt1* or pcDNA-* B4galt1*, and *Cd274* mRNA and PD-L1 protein was analysed by qRT-PCR and western blotting analyses. (G) LLC cells cocultured with activated CD8+ T cells for 48 h were subjected to crystal violet staining and quantified at 570 nm. The ratio of LLC cells to T cells is 1:3. (H) Western blot analysis of PD-L1 expression in LLC cells with or without the knockdown or overexpression of *B4galt1* followed by the treatment with CHX. (I) Western blot analysis of PD-L1 levels in *B4galt1* knock-down cells, *B4galt1* overexpression cells, and tunicamycin (TM) treated cells. * P < 0.05, ** P < 0.01, ****P <0.0001 n.s., not significant. **Figure S4.** (A) Analysis of luciferase activity in LLC cells transfected with the full-length-*Cd724 *promoter and *B4galt1* overexpression plasmid. The results are expressed as the fold change compared with the control cells. (B-C) LLC cells with the knockdown of *Taz*, and *Cd274* mRNA and PD-L1 protein expression were analysed by qRT-PCR and western blotting analyses. (D) Analysis of luciferase activity of *Cd724* in LLC cells transfected with the *Cd724* promoter full-length region and knockdown of *Taz*. The results are expressed as the fold change compared with the control cells. (E) Analysis of luciferase activity of *Cd724* in LLC cells transfected with the *Cd724 *promoter full-length region, along with *B4galt1* pcDNA or *Taz* siRNA. The results are expressed as the fold change compared with the vector cells. (F-G) qRT-PCR and western blotting analyses revealed that *Taz* knockdown mitigated the *B4galt1*-induced upregulation of *Cd274* mRNA and PD-L1 protein levels. * P < 0.05, ** P < 0.01.** Figure S5.** (A) Comparisons of *TAZ* mRNA and TAZ protein levels between the *B4GALT1*-high group and *B4GALT1*-low group (data retrieved from TCGA and TCPA datasets, respectively). (B) Comparisons of T* TAZ* mRNA and TAZ protein levels between the *B4GALT1*-high group and *B4GALT1*-low group (data retrieved from GSE140343 dataset). (C-D) LLC cells were transfected with si-* B4galt1* or pcDNA-* B4galt1*, and *Taz* mRNA and TAZ protein expression was analysed by qRT-PCR and western blotting analyses. (E) Western blot analysis of TAZ expression in LLC cells with or without the knockdown or overexpression of *B4galt1* followed by the treatment with CHX. (F) Western blot analysis of TAZ levels in *B4galt1* knock-down cells, *B4galt1* overexpression cells, and tunicamycin (TM) treated cells. * P < 0.05, ** P < 0.01, n.s., not significant. **Supplementary Table S1. **The sequences of primers and siRNAs.

## Data Availability

The datasets used and/or analyzed during the current study are available from the corresponding author on reasonable request.

## Abbreviations

AIS: Adenocarcinoma in situ
B4GALT1: Beta-1,4-galactosyltransferase 1
BSA: Bovine serum albumin
CCLE: Cancer Cell Line Encyclopedia
CIBERSORT: Cell-type identification by estimating relative subsets of RNA transcripts
cDNA: Complementary DNA
CRISPR: Clustered regularly interspersed short palindromic repeats
CT: Computed tomography
CTL: Cytotoxic T-lymphocyte
CHX: Cycloheximide
DFS: Disease-free survival
DMSO: Dimethyl sulfoxide
DMEM: Dulbecco’s modified Eagle’s medium
EDTA: Ethylenediaminetetraacetic acid
EDU: Ethynyldeoxyuridine
FBS: Fetal bovine serum
FITC: Fluorescein isothiocyanate
GEO: Gene Expression Omnibus
GGO: Ground-glass opacity
GO: Gene ontology
GZMA: Granzyme A
GZMB: Granzyme B
IAC: Invasive adenocarcinoma
KEGG: Kyoto Encyclopedia of Genes and Genomes
LLC: Lewis cells
LUAD: Lung adenocarcinoma
LUL: Left upper lobe
LLL: Left lower lobe
mGGO: Mix ground-glass opacity
MIA: Minimally invasive adenocarcinoma
MPLC: Multiple primary lung cancer
OS: Overall survival
PRF1: Perforin 1
PBMCs: Peripheral blood mononuclear cells
PBS: Phosphate-buffered saline
PD-1: Programmed cell death protein-1
PD-L1: Programmed death-ligand-1
pGGO: Pure ground-glass opacity
qRT–PCR: Quantitative real-time PCR
RIPA: Radioimmunoprecipitation assay
siRNA: Small interfering RNA
SDS-PAGE: Sodium dodecyl sulfate–polyacrylamide gel electrophoresis
RUL: Right upper lobe
RUL: Right upper lobe
TAZ: WW domain-containing transcription regulator 1
TCGA: The Cancer Genome Atlas
TIDE: Tumor Immune Dysfunction and Exclusion
UMAP: Uniform manifold approximation and projection
WB: Western blot
WHO: World Health Organization
