# Supplementary material for: B4GALT1 promotes immune escape by regulating the expression of PD-L1 at multiple levels in lung adenocarcinoma
Source: J Exp Clin Cancer Res. 2023 Jun 12;42:146. doi: 10.1186/s13046-023-02711-3 (PMC10259029; doi:10.1186/s13046-023-02711-3)
Supplement: Supplementary file 1 — Additional file 1: Figure S1. (A-B) qRT-PCR was performed to detect the B4GALT1 mRNA expression after siRNA-mediated knockdown and plasmid-mediated overexpression in A549 and PC9 cells. (C) Western blot was performed to detect the B4GALT1 protein expression. (D) The protein expression levels of CDK4 and Cyclin D1 in control and ectopic B4GALT1-expressing A549 and PC9 cells were determined using western blotting analyses. * P < 0.05, ** P < 0.01. Figure S2. (A) The linear correlations between the B4GALT1 expression and the CD8+ T-cell expression in the TCGA-LUAD dataset. (B) Comparison of TIDE scores between the B4GALT1-high group and B4GALT1-low group. (C) Comparison of overall survival between CTL top (samples above average CTL values among all samples) and CTL bottom (samples below average CTL values among all samples) groups in patients with different B4GALT1 levels. The two-sided Wald test in the Cox-PH regression was applied to compute the association between the CTL level and overall survival, and samples were split by the best separation strategy according to the B4GALT1 expression coefficients in the Cox-PH regression model. ** P < 0.01. Figure S3. (A-B) The differential expression of CD274 in tumours with high expression of B4GALT1 and low expression of B4GALT1 in the CCLE mRNA dataset (A) and the differential expression of PD-L1 in tumours with high expression of B4GALT1 and low expression of B4GALT1 in the TCGA-protein dataset (B). RPKM: reads per kilobase per million mapped reads; RPPA: reverse phase protein array. (C) Representative immunohistochemical images (left panel) and the association between B4GALT1 and PD-L1 level in MPLC tumours (right panel). Scale bars, 200 μm. (D) qRT-PCR was performed to detect the B4galt1 mRNA expression after siRNA-mediated knockdown in LLC cells. (E-F) LLC cells were transfected with si-B4galt1 or pcDNA- B4galt1, and Cd274 mRNA and PD-L1 protein was analysed by qRT-PCR and western blotting analyses. (G) LLC cells cocult [file 13046_2023_2711_MOESM1_ESM.docx]

**Supplemental Information**

**B4GALT1 promotes immune escape by regulating the expression of PD-L1 at multiple levels in lung adenocarcinoma**

Yanan Cui^1#^, Jun Li^1#^, Pengpeng Zhang^2#^, Dandan Yin^3#^, Ziyu Wang^4#^, Wei Wang^2*^, Erbao Zhang^5,6*^, Renhua Guo^1*^

**Contents**

**Supplemental Figure 1**

**Supplemental Figure 2**

**Supplemental Figure 3**

**Supplemental Figure 4**

**Supplemental Figure 5**

**Supplemental Table 1**

**Supplemental Figure 1**

**
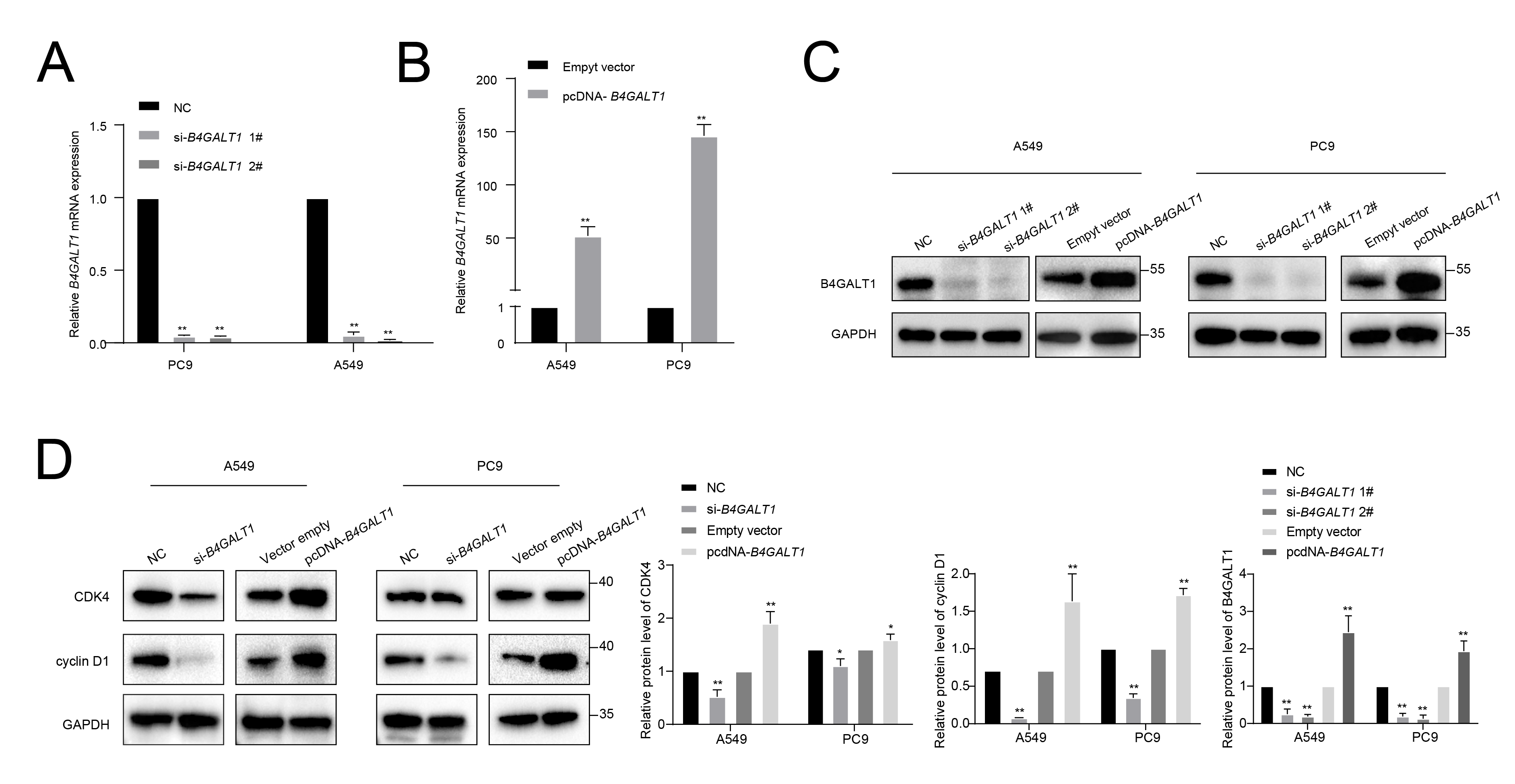
**

**Figure S1.** (A-B) qRT-PCR was performed to detect the *B4GALT1* mRNA expression after siRNA-mediated knockdown and plasmid-mediated overexpression in A549 and PC9 cells. (C) Western blot was performed to detect the B4GALT1 protein expression. (D) The protein expression levels of CDK4 and Cyclin D1 in control and ectopic *B4GALT1*-expressing A549 and PC9 cells were determined using western blotting analyses. *P < 0.05, **P < 0.01.

**Supplemental Figure 2**


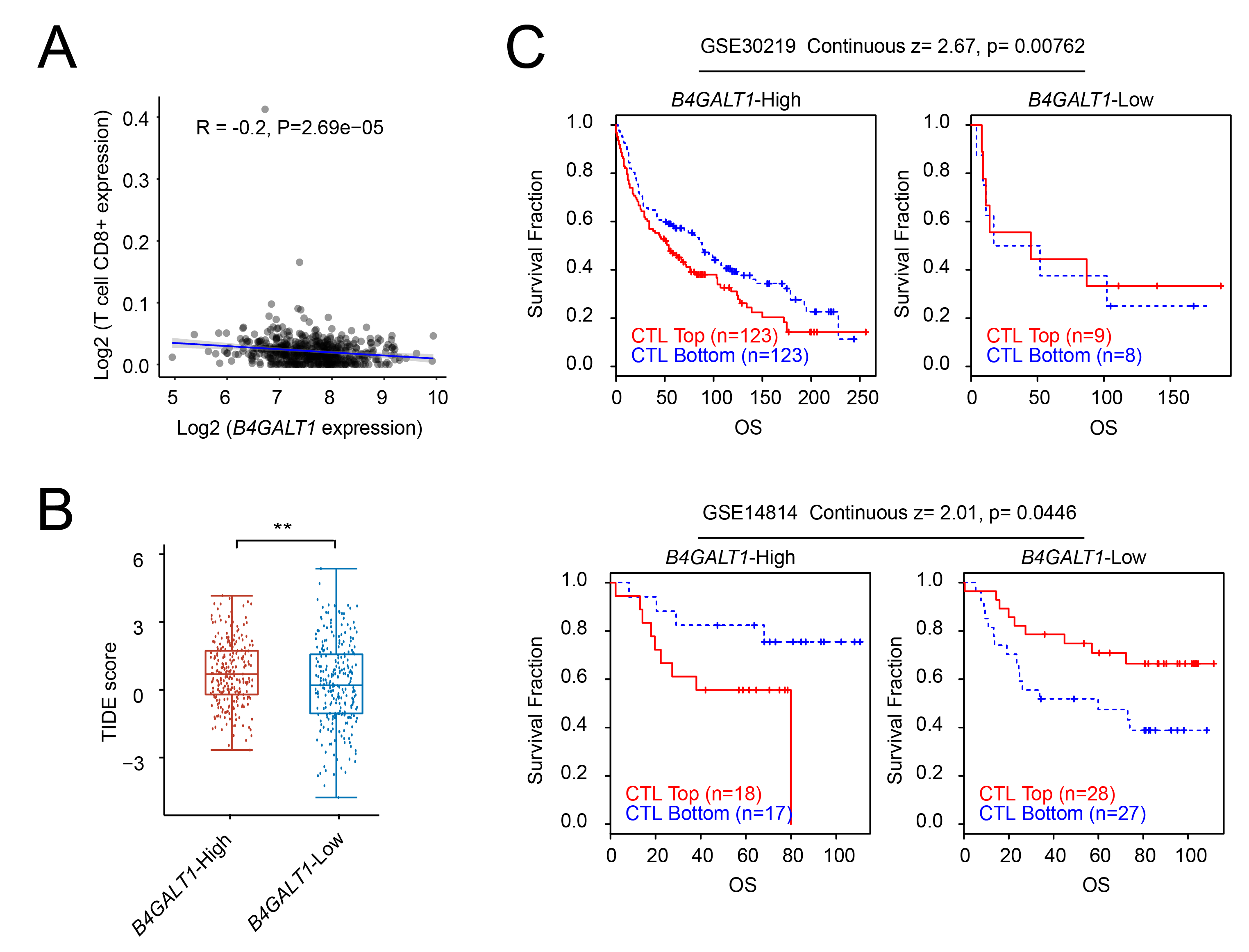


**Figure S2.** (A) The linear correlations between the *B4GALT1* expression and the CD8+ T-cell expression in the TCGA-LUAD dataset. (B) Comparison of TIDE scores between the *B4GALT1*-high group and *B4GALT1*-low group. (C) Comparison of overall survival between CTL top (samples above average CTL values among all samples) and CTL bottom (samples below average CTL values among all samples) groups in patients with different *B4GALT1* levels. The two-sided Wald test in the Cox-PH regression was applied to compute the association between the CTL level and overall survival, and samples were split by the best separation strategy according to the *B4GALT1* expression coefficients in the Cox-PH regression model. **P < 0.01.

**Supplemental Figure 3**

**
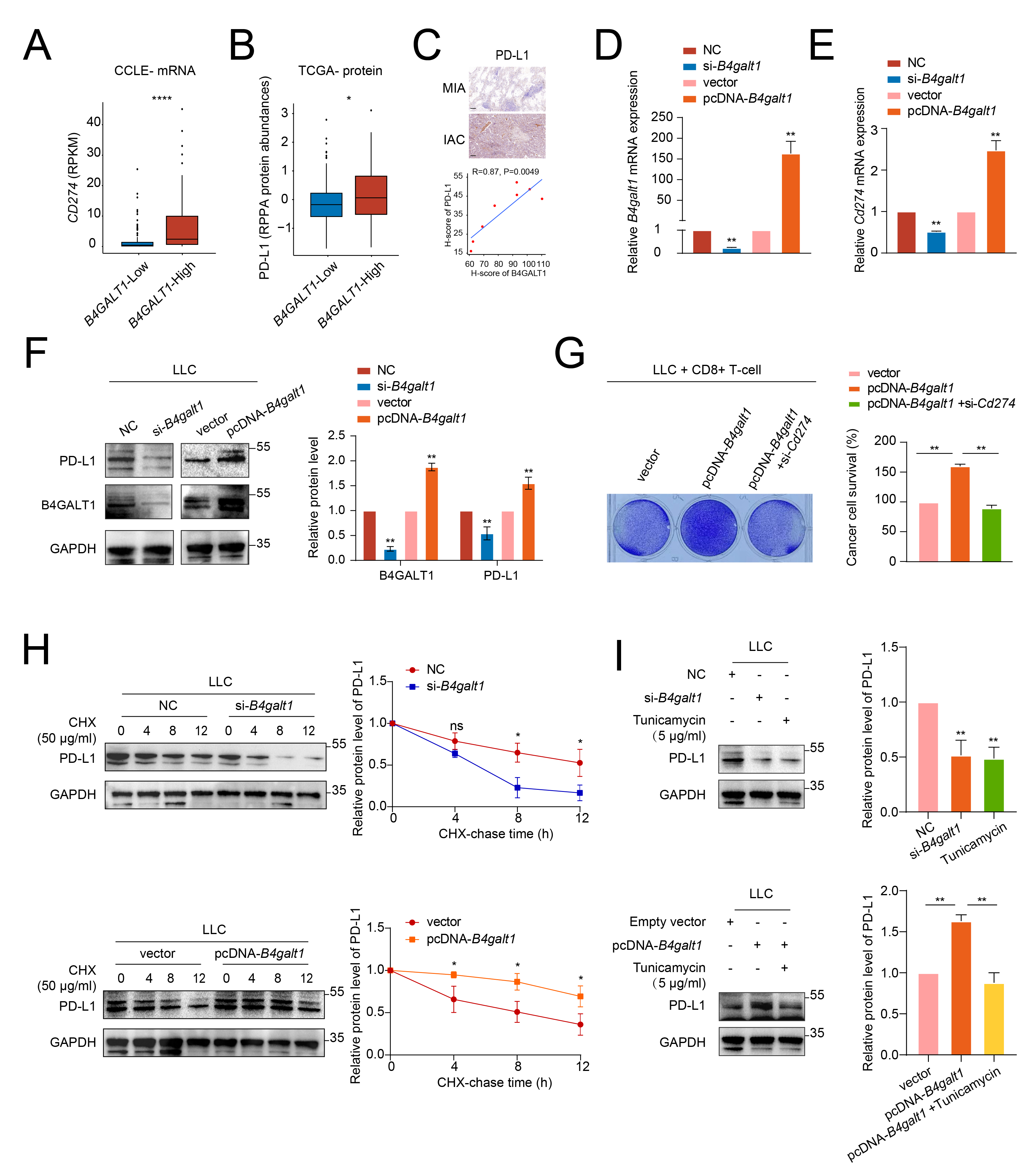
**

**Figure S3.** (A-B) The differential expression of *CD274* in tumours with high expression of *B4GALT1* and low expression of *B4GALT1* in the CCLE mRNA dataset (A) and the differential expression of PD-L1 in tumours with high expression of *B4GALT1* and low expression of *B4GALT1* in the TCGA-protein dataset (B). RPKM: reads per kilobase per million mapped reads; RPPA: reverse phase protein array. (C) Representative immunohistochemical images (left panel) and the association between B4GALT1 and PD-L1 level in MPLC tumours (right panel). Scale bars, 200 μm. (D) qRT-PCR was performed to detect the *B4galt1* mRNA expression after siRNA-mediated knockdown in LLC cells. (E-F) LLC cells were transfected with si-*B4galt1* or pcDNA- *B4galt1*, and *Cd274* mRNA and PD-L1 protein was analysed by qRT-PCR and western blotting analyses. (G) LLC cells cocultured with activated CD8+ T cells for 48 h were subjected to crystal violet staining and quantified at 570 nm. The ratio of LLC cells to T cells is 1:3. (H) Western blot analysis of PD-L1 expression in LLC cells with or without the knockdown or overexpression of *B4galt1* followed by the treatment with CHX. (I) Western blot analysis of PD-L1 levels in *B4galt1* knock-down cells, *B4galt1* overexpression cells, and tunicamycin (TM) treated cells. *P < 0.05, **P < 0.01, ****P<0.0001 n.s., not significant.

**Supplemental Figure 4**


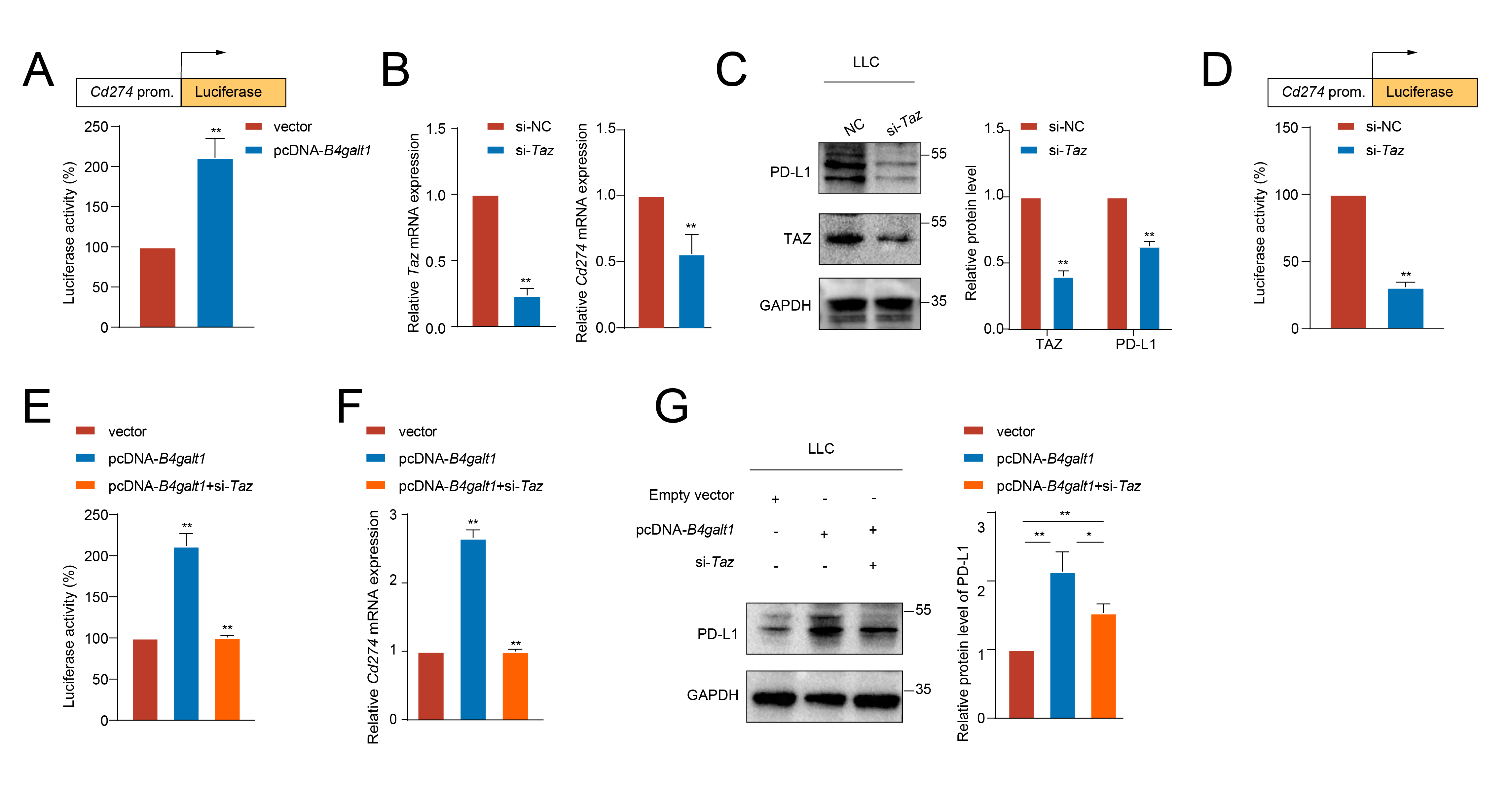


**Figure S4.** (A) Analysis of luciferase activity in LLC cells transfected with the full-length-*Cd724* promoter and *B4galt1* overexpression plasmid. The results are expressed as the fold change compared with the control cells. (B-C) LLC cells with the knockdown of *Taz*, and *Cd274* mRNA and PD-L1 protein expression were analysed by qRT-PCR and western blotting analyses. (D) Analysis of luciferase activity of *Cd724* in LLC cells transfected with the *Cd724* promoter full-length region and knockdown of *Taz*. The results are expressed as the fold change compared with the control cells. (E) Analysis of luciferase activity of *Cd724* in LLC cells transfected with the *Cd724* promoter full-length region, along with *B4galt1* pcDNA or *Taz* siRNA. The results are expressed as the fold change compared with the vector cells. (F-G) qRT-PCR and western blotting analyses revealed that *Taz* knockdown mitigated the *B4galt1*-induced upregulation of *Cd274* mRNA and PD-L1 protein levels. *P < 0.05, **P < 0.01.

**Supplemental Figure 5**


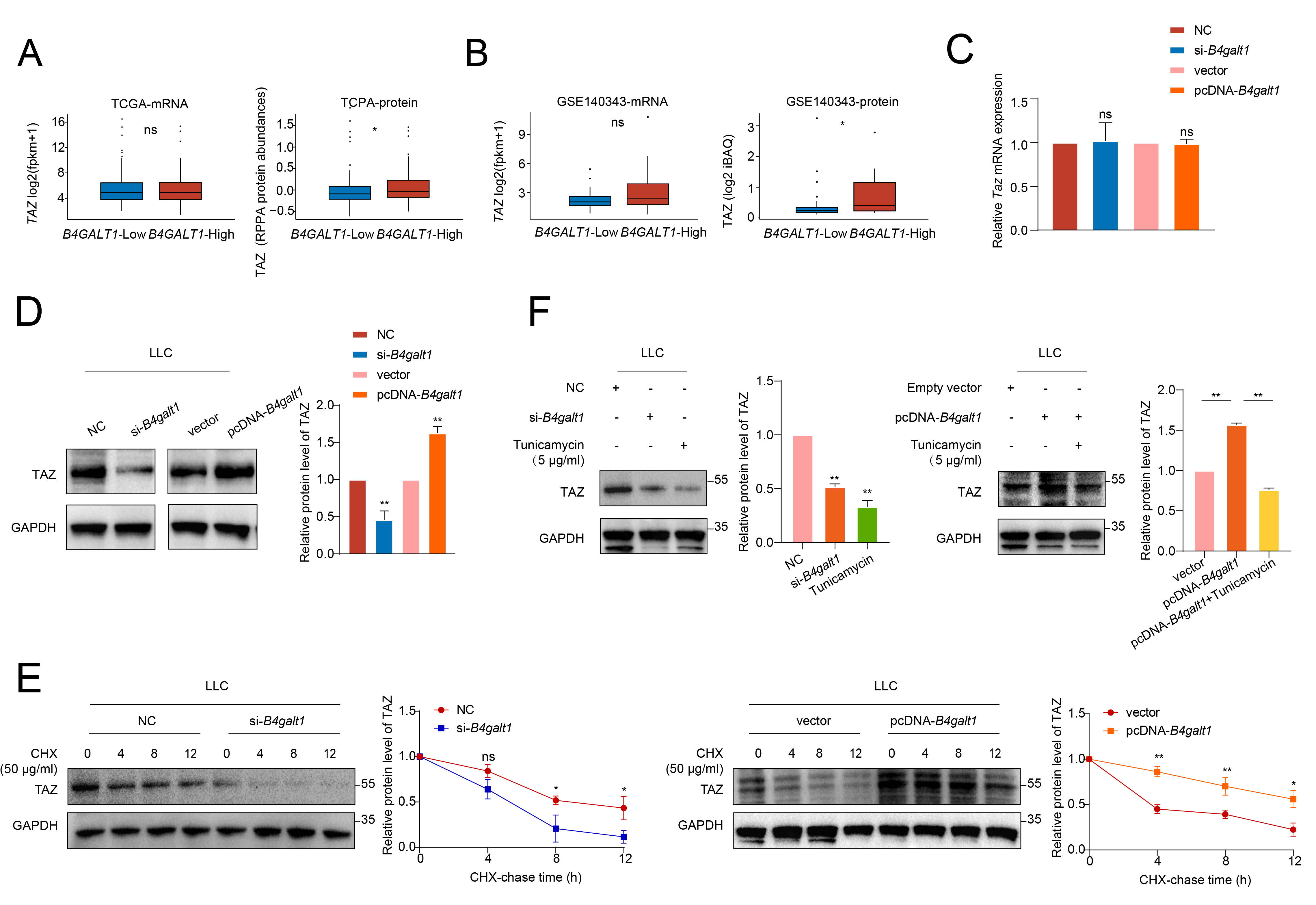


**Figure S5.** (A) Comparisons of *TAZ* mRNA and TAZ protein levels between the *B4GALT1*-high group and *B4GALT1*-low group (data retrieved from TCGA and TCPA datasets, respectively). (B) Comparisons of T *TAZ* mRNA and TAZ protein levels between the *B4GALT1*-high group and *B4GALT1*-low group (data retrieved from GSE140343 dataset). (C-D) LLC cells were transfected with si- *B4galt1* or pcDNA- *B4galt1*, and *Taz* mRNA and TAZ protein expression was analysed by qRT-PCR and western blotting analyses. (E) Western blot analysis of TAZ expression in LLC cells with or without the knockdown or overexpression of *B4galt1* followed by the treatment with CHX. (F) Western blot analysis of TAZ levels in *B4galt1* knock-down cells, *B4galt1* overexpression cells, and tunicamycin (TM) treated cells. *P < 0.05, **P < 0.01, n.s., not significant.

**Supplementary Table S1. The sequences of primers and siRNAs.**

| Gene | | Sequence (5’-3’) |
| --- | --- | --- |
| qRT-PCR primers | | |
| *GAPDH* | Forward primer | AGCCACATCGCTCAGACAC |
|  | Reverse primer | GCCCAATACGACCAAATCC |
| *B4GALT1* | Forward primer | CTAGCAACTTGACCTCGGT |
|  | Reverse primer | GGGTTCTGCTTTGCC |
| *CD274* | Forward primer | TGACCTACTGGCATTTGCTG |
|  | Reverse primer | TGCAGCCAGGTCTAATTGTTT |
| *TAZ* | Forward primer | TGGACCAAGTACATGAACCACC |
|  | Reverse primer | TCCACAGCCGACTTGTTCTC |
| *Gapdh* (mouse) | Forward primer | AGGTCGGTGTGAACGGATTTG |
|  | Reverse primer | GGGGTCGTTGATGGCAACA |
| *B4galt1* (mouse) | Forward primer | AAGAACTGACGCCCTCATGG |
|  | Reverse primer | AGCTGAGCCTAAGGATCCCA |
| *Taz* (mouse) | Forward primer | CCGTGCACAACAAGGAAGTG |
|  | Reverse primer | TCAGCAATCAGCCGTCCAAT |
| *Cd274* (mouse) | Forward primer | GCTCCAAAGGACTTGTACGTG |
|  | Reverse primer | TGATCTGAAGGGCAGCATTTC |
| Sequences for siRNAs | | |
| si-B4GALT1 1# | sense sequence | ACCUCAAGUACUGGCUAUAUUTT |
|  | antisense sequence | AAUAUAGCCAGUACUUGAGGUTT |
| si-B4GALT1 2# | sense sequence | GAUACCCAUUGUAUACCCAAATT |
|  | antisense sequence | UUUGGGUAUACAAUGGGUAUCTT |
| si-B4GALT1 (mouse) | sense sequence | GGUGUUGGAUGUACAGAGATT |
|  | antisense sequence | UCUCUGUACAUCCAACACCTT |
| si-TAZ | sense sequence | CAUCUGGAACCUGAAGUUGAUTT |
|  | antisense sequence | AUCAACUUCAGGUUCCAGAUGTT |
| si-TAZ (mouse) | sense sequence | GCAGAACAGCUCCACAAUCAUTT |
|  | antisense sequence | AUGAUUGUGGAGCUGUUCUGCTT |
| si-CD274 | sense sequence | GCCGAAGUCAUCUGGACAATT |
|  | antisense sequence | UUGUCCAGAUGACUUCGGCTT |

Abbreviations: siRNA, small interfering RNA; qRT-PCR, quantitative real-time polymerase chain reaction.
